# Effect of Paracetamol versus Ibuprofen in Adenotonsillectomy

**DOI:** 10.22038/IJORL.2021.56501.2945

**Published:** 2021-11

**Authors:** Fatemeh Mirashrafi, Fatemeh Tavakolnejad, Behrooz Amirzargar, Arezou Abasi, Amin Amali

**Affiliations:** 1 *Otorhinolaryngology Research Center,* *Tehran University of Medical Sciences, Tehran, Iran.*

**Keywords:** Adenotonsillectomy, Paracetamol, Ibuprofen

## Abstract

**Introduction:**

The present study aimed to compare the effects of paracetamol and ibuprofen on pain, bleeding, nausea, and vomiting following adenotonsillectomy in children.

**Materials and Methods:**

This was a prospective, double-blinded, randomized clinical trial. Block randomization was used to assign 50 patients to two groups of paracetamol and ibuprofen. In the paracetamol group, subjects received 15 mg/kg oral paracetamol 30 minutes before the induction of anesthesia, followed by the same dosage every 6 hours postoperatively. Meanwhile, the ibuprofen-treated group took 10 mg/kg oral ibuprofen 30 minutes before and every 6 hours after the operation. The subjects in both groups received the medication for three postoperative days. The postoperative pain score was assessed 6 hours after the surgery and during the second and the third postoperative days. Nausea and vomiting episodes were recorded in the first postoperative day and first postoperative week.

**Results:**

Based on the results, intraoperative and postoperative bleeding in both groups was not significantly different. The mean score of pain in the first postoperative day (6 hours after the surgery) and the second and the third postoperative days did not show any statistical difference. The ibuprofen group experienced fewer vomiting episodes, compared to the paracetamol group during the first postoperative day (P=0.011). Vomiting episodes in the first postoperative week did not illustrate any significant difference.

**Conclusion:**

As evidenced by the results of the current study, Ibuprofen had the same effect on the alleviation of postoperative pain, caused fewer vomiting episodes, and did not cause excessive bleeding as an NSAID. Therefore, oral administration of ibuprofen is suggested for pain relief and management of other complications following adenotonsillectomy in children.

## Introduction

Tonsillectomy is one of the most common surgical procedures in children (1,2), and recurrent throat infections and sleep-disordered breathing are the main indications of this procedure. Since it is performed on a large scale, tonsillectomy can be considered a minor procedure with minor complications. Nonetheless, postoperative severe pain, bleeding, nausea and vomiting can make tonsillectomy a challenging procedure (3-5). Pain is an inevitable complication; moreover, the incidence of nausea and vomiting is considered to be 40%-70% (6). In addition, the risk of bleeding varies between 1%-5% (3). 

These complications delay the postoperative oral fluid intake, which leads to dehydration. The management of these complications helps to maintain oral fluid intake and avoid a prolonged hospital stay, leading to a speedy return to normal activities. Opioids, as powerful analgesic drugs, are not favorable due to opioid-induced respiratory depression, sedation, as well as the high incidence of nausea and vomiting. Non-steroidal anti-inflammatory drugs (NSAIDs) and paracetamol are good alternatives to opioids. Based on the literature, NSAIDs (other than Ketorolac) are not associated with increased postoperative bleeding. It seems that these drugs can be safely used for the management of pain after tonsillectomy (7-9). According to the Association of Pediatric Anesthetists of Great Britain and Ireland (APA) guidelines on the prevention of postoperative vomiting, the administration of dexamethasone will help control nausea and vomiting in children undergoing tonsillectomy (10). The present randomized, double-blinded clinical study aimed to compare the effects of paracetamol and ibuprofen in pain relief and management of other complications following tonsillectomy in children aged 4-10 years old.

## Materials and Methods

A prospective, double-blinded, randomized trial (IRCT2013071814047N1) was initiated to investigate the comparative effects of paracetamol and ibuprofen on pain relief following adenotonsillectomy. 

Between March 2015 and February 2017, consecutive children who were candidates of elective adenotonsillectomy in Children’s Medical Centre were assessed for eligibility. The inclusion criteria were as follows: the age range of 4-10 years old, American Society of Anesthesiologists classes I or II, no allergy to NSAIDs, no history of coagulopathy, no renal and hepatic disorder, no severe asthma, no family history of coagulopathy, and no evidence of mental retardation. According to a similar article and sample size formula, a total of 52 patients were included in the study. Block randomization using a block size of 4 was used to assign patients to two groups of paracetamol and ibuprofen. A nurse performed the randomization by opening the envelope which contained the randomization number (1 or 2). There were two syrup bottles with identical shapes which were labeled as 1 or 2 (paracetamol and ibuprofen groups, respectively). In the paracetamol group, subjects received 15 mg/kg oral paracetamol 30 minutes before the induction of anesthesia, followed by the same dosage every 6 hours postoperatively. Meanwhile, the ibuprofen-treated group took 10 mg/kg oral ibuprofen 30 minutes before and every 6 hours after the operation.

The subjects in both groups received the medication for three postoperative days. The patients and parents were blinded to the type of medications that had been prescribed and the surgeon. The Wong-Baker visual analog pain scale (VAS) graded from 0-10 was used for the assessment of pain. In the preoperative visit, all patients and their parents were trained on the use of the VAS. The patients who reported a missing dose of medications were excluded from the study. All procedures regarding human subjects were conducted in accordance with the declaration of Helsinki. The Ethics Committee of Tehran University of Medical Sciences approved the study protocol. Written informed consent was acquired from all parents prior to enrolment.

General anesthesia was induced using sevoflurane 8%. Endotracheal intubation was facilitated by atracurium 5 mg/kg, thiopental 5 mg/kg, and fentanyl 1 µg/kg. Anesthesia was maintained using 1.5%-2% sevoflurane and 50% N2O. Dexamethasone (as an antiemetic drug) was administered in a single intraoperative dose (0.5 mg/kg) for all patients. Adenoidectomy is performed using a curve adenoid curette, and tonsillectomy is carried out using an extracapsular technique with a cold knife and bipolar cautery. All the operations were performed by the same surgeon. Intraoperative bleeding was assessed by the number of used gauzes and the amount of blood in the suction pump bottle. The occurrence of postoperative bleeding was recorded in the first postoperative day and first postoperative week.

The postoperative pain score was assessed 6 hours after the surgery (inpatient setting), as well as during the second and the third postoperative days (via a questionnaire recorded by patients). Nausea and vomiting episodes were recorded in the first postoperative day and first postoperative week (via a questionnaire). Moreover, the time that the patient tolerated the usual diet and the time that they returned to usual activity was recorded (via a questionnaire). The data were analyzed in SPSS software (version 19.0) using t-test, chi-square test, and Mann-Whitney U-test for group, frequency, and pain score comparison, respectively. Continuous variables were expressed as Mean±SD. A p-value of less than 0.05 was considered statistically significant.

## Results

**Fig 1:**
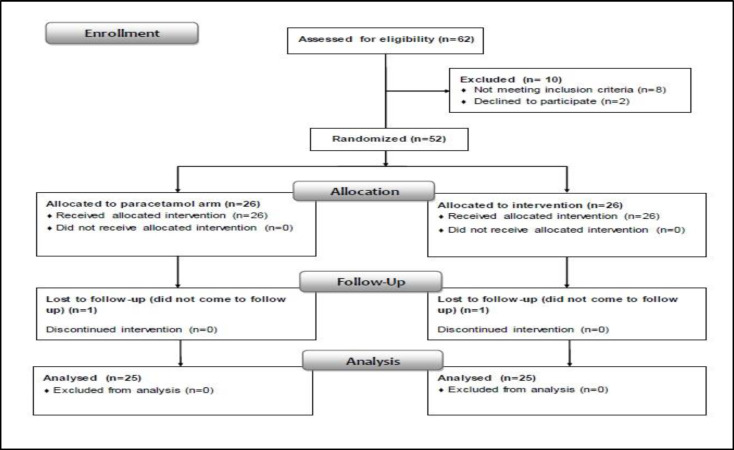
CONSORT flowchart of the study

A total of 52 patients met the criteria to participate in this study, out of whom two patients (n=1 from each group) were lost to follow-up. The analysis was performed on the remaining 50 subjects (paracetamol=25, ibuprofen=25) (Figure 1).

The indications for adenotonsillectomy included: recurrent tonsillitis (52%), sleep apnea (36%), and obstructive adenotonsillar hypertrophy (12%).

Based on the results, 24 (48%) patients were female and 26 (52%) cases were male, 13 (52%) patients in the paracetamol group and 13 (52%) subjects in the ibuprofen group were male (P value=1.000). The mean age scores of all participants, the paracetamol group, and the ibuprofen group were reported as 6.94±1.83 6.8±1.75, and 7.08±1.93 years, respectively (P=0.595). The mean body weight scores of all the patients, the paracetamol group, and the ibuprofen group were obtained at 24.38±4.12, 24.04±4.08, and 24.72±4.22 kg, respectively (P=0.566). No significant differences were observed between the two groups in age, gender, and mean body weight. 

Mean scores of intraoperative bleeding of all patients, paracetamol group, and ibuprofen group were 3.30±0.81, 3.20±0.73, and 3.42±0.89 ml/kg, respectively (P=0.36). Five patients experienced postoperative bleeding in the first postoperative day (two patients in the paracetamol group and three patients in the ibuprofen group (P=1.00). Bleeding was controlled in the ward, and no surgical intervention was required. No bleeding was recorded in the first postoperative week. The mean score of pain in the first postoperative day (6 hours after the surgery), as well as the second and the third postoperative days, did not show any statistical difference (Table.1). 

There was no patient who required additional analgesia due to insufficient pain control in both groups.

**Table 1:** Mean of pain scores during postoperative days

**Mean scores of pain**	**All of ** **the ** **patients**	**Paracetamol group**	**Ibuprofen group**	**P** **-** **value**
First postoperative day	6.08	6	6.16	0.774
Second postoperative day	4.08	4.16	4	0.779
Third postoperative day	1.92	2.04	1.80	0.705
				

The ibuprofen group experienced fewer vomiting episodes (0.24±0.52), compared to the paracetamol group (0.72±0.73) during the first postoperative day. This finding was statistically significant (P= 0.011). Vomiting episodes in the first postoperative week did not demonstrate any significant difference. The mean postoperative day for the initiation of oral fluid, as well as semi-solid and solid intake, in both groups was not significantly different (Table 2). 

**Table 2:** Mean postoperative day for initiation of oral intake

**Mean postoperative day**	**All of ** **the ** **patients**	**Paracetamol group**	**Ibuprofen group**	**P** **-** **value**
Oral fluid	1.20	1.04	1.36	0.092
Semi-solid	2.36	2.60	2.12	0.10
Solid	3.70	3.92	3.48	0.25

## Discussion

This randomized clinical trial study aimed to compare the effect of acetaminophen and ibuprofen on the management of pain and other postoperative complications following adenotonsillectomy. Along with other studies, adenotonsillectomy, in the present research, is mainly performed due to recurrent throat infection, sleep apnea, and obstructive adenotonsillar hypertrophy (11,12). Since eligible patients for this procedure are mostly children aged 4-10 years old, postoperative pain and other complications make this commonly performed procedure more challenging. In the past decades, opioids were largely administered as analgesics for pain management; nonetheless, their adverse effects limited their administration (3,7). Other active analgesics, such as acetaminophen and NSAIDs, are currently being more widely used (13).

Acetaminophen was orally administered in the acetaminophen group since this route is more effective. A repeated dosage of acetaminophen and ibuprofen (every 6 hours) was administered for maintaining better pain management. As described by Humanen et al., a single dosage of analgesic is not sufficient for postoperative pain relief (10). The results of the current study reinforced those obtained by Merry et al. who reported that acetaminophen and ibuprofen have no significant difference in the alleviation of postoperative pain (14). However, contrary to our findings, Mahgoobifard et al. reported significantly lower pain with acetaminophen, in comparison with ibuprofen (5). This discrepancy can be explained according to the study by Hamunen et al. who indicated that when considering the equipotency of drugs, larger group size is mandatory for revealing smaller differences between analgesic effects of two drugs (4). Therefore, further studies with larger sample sizes will help in elucidating this controversy. As evidenced by the obtained results, intra- and postoperative bleeding was not different in the acetaminophen and ibuprofen groups; moreover, ibuprofen did not cause excessive bleeding as an NSAID. This is in keeping with a report by Isaacson and other studies (3,13-16). It can be concluded that ibuprofen, as an effective analgesic, is a safe drug regarding the concerns about hemostasis difficulty caused by ketorolac (12).

  The mechanism of post-tonsillectomy nausea and vomiting can be ascribed to blood stasis in the pharynx, as well as oropharyngeal sensitivity due to surgical manipulation and tracheal intubation. According to the literature (6-11), the incidence of nausea and vomiting ranges from 40%-70%. In the present study, the overall incidence of nausea and vomiting was 38%. This was significantly lower in the ibuprofen group. As reported by White et al., NSAIDs reduce the incidence of nausea and vomiting (9). This finding may be attributed to the effect of NSAIDs on the reduction of inflammatory mediators and sensitivity of the surgical site.Among the notable limitations of the present study, we can refer to the use of VAS for postoperative pain measurement in children. In our study, the patients were children aged 4-10 years old, and in this age group, VAS is less reliable for precise pain measurement (14).

## Conclusion

 As illustrated by the results of the present study, Ibuprofen had the same effect on the alleviation of postoperative pain, caused fewer vomiting episodes, and did not cause excessive bleeding as an NSAID. Therefore, oral administration of ibuprofen is suggested for pain relief and management of other complications following adenotonsillectomy in children.
